# Consequences of cure: subsequent therapy-related cancers

**DOI:** 10.18632/oncotarget.23003

**Published:** 2017-12-06

**Authors:** Lucie M. Turcotte, Joseph P. Neglia

**Affiliations:** University of Minnesota Medical School, Department of Pediatrics, Minneapolis, MN, USA

**Keywords:** survivorship, radiation, subsequent neoplasm

Survival following a childhood cancer diagnosis has improved dramatically over the last five decades [1]. While “cure” was felt to be impossible for childhood malignancies in the 1940s, it is now an expectation for over 80% of children who receive new cancer diagnoses [2]. Despite this very real victory, cure comes with significant costs to many survivors. The field of cancer survivorship has rapidly expanded as recognition of the myriad late health consequences, or late effects, experienced by survivors has increased. Subsequent neoplasms (SNs), or new primary neoplasms occurring after an initial childhood cancer diagnosis, are associated with the highest rates of mortality among these late effects and contribute greatly to morbidity among survivors. SNs are highly associated with previous therapeutic exposures. Specifically, alkylating agent and epipodophyllotoxin exposure are associated with the development of treatment-related acute leukemias and myelodysplasia, and therapeutic radiation exposure is associated with subsequent solid tumors and non-melanoma skin cancers. Although the latency from the time of cancer treatment to SN development can be quite variable, it is typically quite early (<3 to 8 years) for treatment-related leukemia/myelodysplasia and much later for solid tumors (>10 years).

Over time, our understanding about SN etiology and dose-response relationships have improved. Chemotherapeutic dose thresholds have been identified that are associated with greatest risk and as a result, therapies have been refined when possible. In certain pediatric malignancies, such as acute lymphoblastic leukemia (ALL), Hodgkin lymphoma (HL) and Wilms tumor, where certain sub-groups have achieved long-term survival exceeding 90%, efforts have turned toward making minor therapeutic modifications with the ultimate goal of reducing risk for late effects while maintaining current outcomes. Among ALL patients, cranial radiation has been eliminated from treatment for nearly all patients. Similarly, use of radiation in patients with HL has moved toward a risk-stratified approach, only incorporating involved-field therapeutic radiation into the treatment of the highest risk or slower responding patients. Use of abdominal radiation has decreased among Wilms tumor patients as well.

Although it has been assumed that these therapeutic modifications would lead to improved outcomes and decreased toxicities for children with cancer, both in the short and long-term, these benefits had not been shown until recently. Armstrong et al. demonstrated a decrease in late-mortality among the Childhood Cancer Survivor Study (CCSS) cohort of 34,033 5-year survivors of childhood cancer diagnosed before 21 years of age between 1970 to 1999. Fifteen-year mortality decreased from 12.4% for those diagnosed in the 1970s to 6.0% for those diagnosed in the 1990s (*p_trend_* <0.001) and these reductions were partially attributable to reduced mortality from SNs [3].

Recently, we hypothesized that not only was late-mortality secondary to SNs improving over time, but that the actual incidence of and risk for SNs was decreasing as well. We further hypothesized that these reductions would be associated with decreasing therapeutic exposures. To address these hypotheses, we analyzed detailed treatment and SN data from the CCSS and reported SNs by decade of diagnosis. Our findings were published earlier this year in “JAMA” [4]. Among 23,603 survivors, the percentage of individuals receiving therapeutic radiation decreased from 77% in the 1970s to 33% in the 1990s, as did the median dose delivered (30 Gy in 1970s vs. 26 Gy in the 1990s). During this timeframe, the proportion of individuals receiving chemotherapy increased. Fifteen-year cumulative incidence of subsequent malignant neoplasms decreased with advancing treatment decades (2.1%, 95% CI 1.7%-2.4% for 1970s vs. 1.3%, 95% CI 1.1%-1.5% for 1990s) and standardized incidence ratios (SIRs) declined over treatment decades for survivors with attained ages ≥20 years. Relative rates of SNs decreased with each 5-year increment and radiation dose reductions were significantly associated with reduced risk for SNs. This was the first study to demonstrate the positive impact of therapeutic modifications on the incidence of SNs.

In contrast to our findings, others have not shown reductions in SN risk over time, despite similar reductions in therapeutic radiation exposure. A recent publication on Dutch childhood cancer survivors (DCOG-LATER) reported similar 15-year cumulative incidence and risk of SNs, regardless of treatment decade [5]. Similarly, in an analysis of HL survivors diagnosed as adolescents or adults in the Netherlands diagnosed between 1965- to 2000, the risk of subsequent solid cancers did not decrease over time [6]. These findings indicate other factors at play beyond radiation in the development of SNs among survivors.

It is clear that we must further expand our knowledge regarding the underlying pathobiology of SNs. Over 15 million people in the US are cancer survivors [7] and deserve focused, informed care. Additionally, multiple novel therapies have been introduced in recent years with unique mechanisms of action, such as immunotherapies and targeted therapies, and these may alter the immune system in ways that previous therapies have not and ultimately may alter the risk for SNs. The role of unique genetic variants in predicting early and late treatment toxicities is emerging as an exciting area of research opportunity, as are other host biological characteristics such as epigenetic modifications, telomere maintenance and immune function. By broadening our understanding of these risks, we may identify modifiable characteristics that could ultimately lead to improved long-term outcomes for this ever-increasing patient population.
